# Generation Mechanism of Anisotropy in Mechanical Properties of WE43 Fabricated by Laser Powder Bed Fusion

**DOI:** 10.3390/mi15080976

**Published:** 2024-07-30

**Authors:** Jingfei Bai, Qiulin Wang, Zhengxing Men, Wen Chen, Huanjie Huang, Chen Ji, Yong Li, Liang Wang, Liang Zhu, Kun Li, Qing Su

**Affiliations:** 1 Chengdu Aeronautic Polytechnic, Chengdu 610100, China; bjf@cap.edu.cn (J.B.); 1500007@cap.edu.cn (Q.W.); menzhengxing@cap.edu.cn (Z.M.); 1400010@cap.edu.cn (Y.L.); wangliang@cap.edu.cn (L.W.); 2 College of Mechanical and Vehicle Engineering, Chongqing University, Chongqing 400044, China; w-chen@cqu.edu.cn (W.C.); huanjie.h@stu.cqu.edu.cn (H.H.); 20220701068@cqu.edu.cn (C.J.); zhuliang@cqu.edu.cn (L.Z.); 3 Chongqing Key Laboratory of Metal Additive Manufacturing (3D Printing), Chongqing University, Chongqing 400044, China; 4 State Key Laboratory of Mechanical Transmission for Advanced Equipment, Chongqing University, Chongqing 400044, China; 5 Material Corrosion and Protection Key Laboratory of Sichuan Province, Zigong 643000, China; suqingwl@163.com

**Keywords:** laser powder bed fusion, WE43 magnesium alloy, anisotropy, generation mechanism

## Abstract

At present, no consensus has been reached on the generation mechanism of anisotropy in materials fabricated by laser powder bed fusion (LPBF), and most attention has been focused on crystallographic texture. In this paper, an analysis and test were carried out on the hardness, defect distribution, residual stress distribution, and microstructure of WE43 magnesium alloy fabricated by LPBF. The results indicate that LPBF WE43 exhibits obvious anisotropy—the hardness HV of X–Z surface (129.9 HV on average) and that of Y–Z surface (130.7 HV on average) are about 33.5% higher than that of X–Y surface (97.6 HV on average), and the endurable load is smaller in the stacking direction Z compared to the X and Y directions. The factors contributing more to the anisotropy are listed as follows in sequence. Firstly, the defect area of the X–Y projection surface is about 13.2% larger than that of the other two surfaces, so this surface shows greatly reduced mechanical properties due to the exponential relationship between the material strength and the number of defects. Secondly, for laser scanning in each layer/time, the residual stress accumulation in the Z direction is higher than that in the X and Y directions, which may directly reduce the mechanical properties of the material. Finally, more fine grains are distributed in X–Z and Y–Z surfaces when comparing them with those in an X–Y surface, and this fine-grain strengthening mechanism also contributes to the anisotropy. After T5 aging heat treatment (250 °C/16 h), a stronger crystallographic texture is formed in the <0001> direction, with the orientation density index increasing from 10.92 to 21.38, and the anisotropy disappearing. This is mainly caused by the enhancement effect of the texture in the <0001> direction on the mechanical properties in the Z direction cancelling out the weakening effect of the defects in the X–Y surface in the Z direction.

## 1. Introduction

As a high-strength, thermal-resistant, magnesium alloy, WE43 is supplemented with rare earth elements such as Y and Nd [1,2], enabling it to hinder matrix diffusion and to generate a new stable phase during heat treatment. With nail dislocation at high temperatures and grain boundary movement, the high temperature properties of magnesium alloy are improved and have gained popularity in aerospace and other fields [3,4,5]. Due to its extremely fast solidification rate, laser powder bed fusion (LPBF) technology can obviously change the solid solution state and super-saturation of the formed alloy solute. Compared with the equilibrium state, the solid solubility of the LPBF-based WE43 alloy solute is enhanced at the same temperature, forming a feature of super-solid solution of the element [6,7,8,9]. As a result, some mechanical properties of the magnesium alloy with additive manufacturing can meet the standard for mechanical properties of the magnesium alloy prepared with traditional methods [10,11,12].

It has been found that materials formed by LPBF have differences in mechanical properties in different directions. Raiyan S et al. [13] employed LPBF technology to manufacture lightweight Fe–Mn–Al–C steel, whose average strength and elongation are 1.3 GPa and 36% in the direction parallel to its construction direction, and 1.1 GPa and 20% in the direction perpendicular to its construction direction, showing obvious anisotropy. The biomaterial Ti-6Al-7Nb alloy manufactured by Milaege D et al. [14] with LPBF technology also exhibits distinct anisotropy, which complicates the loading state of the implant and does not meet the minimum requirements specified in standards such as ISO 5832–11 [15]. After analysis and tests, the samples arranged at 90° relative to the building platform performed the best under fatigue load, meeting all the requirements related to monotonic loading in the standards. From this perspective, anisotropy can limit the range of material use.

To reveal the generation mechanism of anisotropy and to provide a theoretical basis for the adjustment mechanism of mechanical properties and other properties, Jiang W et al. [16] prepared Al–Mn–Sc alloy by adopting selected laser melting (SLM). Due to the local re-melting, competitive growth mechanism and specific heat flow direction in the previous layer, the <113>//BD preferentially oriented cylindrical grains formed along the construction direction. For the already constructed Al–Mn–Sc alloy, the equiaxed grains with <112>//TD and weak <212>//TD textures were dominant in transverse direction, accompanied by mechanical anisotropy. After heat treatment at 300 °C for 5 h, the mechanical anisotropy decreased. Jiang W et al. [17], by introducing an in-situ EBSD stretching method and eliminating the influence of size and length–diameter ratio of grains, revealed the orientation-induced anisotropy coefficient Ka related to grain rotation caused by deformation. When comparing this with the traditional Taylor factor M mechanical properties model, it was discovered that the orientations of <001> in initial treatment and <102> in heat treatment contribute the most yield strength anisotropy. By introducing Ka, mechanical anisotropy can be predicted more accurately. By establishing a reduced-order crystal plastic finite element (CPFE) model, Liu Yang et al. [18] studied the influence of microstructure morphology and crystallographic texture on the mechanical anisotropy of SLM Ti-6Al-4V. The graded and equiaxed CPFE models with the same crystallographic texture exhibited the same mechanical anisotropy. At the particle scale, the significance of crystallographic anisotropy varied with the crystal orientation. Crystallographic texture was deemed the main controlling variable of mechanical anisotropy of Ti-6Al-4V formed by LPBF.

At present, no consensus has been reached on the generation mechanism of anisotropy caused by LPBF [19,20,21,22,23]. With most attention focusing on crystallographic texture, the distribution of defects, residual stress, grain size and other aspects attract less attention. In this paper, by combining with a T5 aging treatment experiment of LPBF-based WE43 alloy, and eliminating the anisotropy therein, the specific causes of anisotropy in LPBF-based WE43 alloy were studied, providing a reference for the adjustment mechanism of mechanical properties of LPBF-based WE43 alloy.

## 2. Materials and Methods

### 2.1. Materials

The experimental material is WE43 spherical powder of rare earth magnesium alloy prepared by rotating plasma atomization, with a particle size of 30~50 μm and good fluidity. Its chemical composition is exhibited in Table 1. The powder was put into a vacuum drying furnace for 8 h at a temperature of 40 °C, aiming to remove the moisture.

### 2.2. Methods

**Figure 1:**
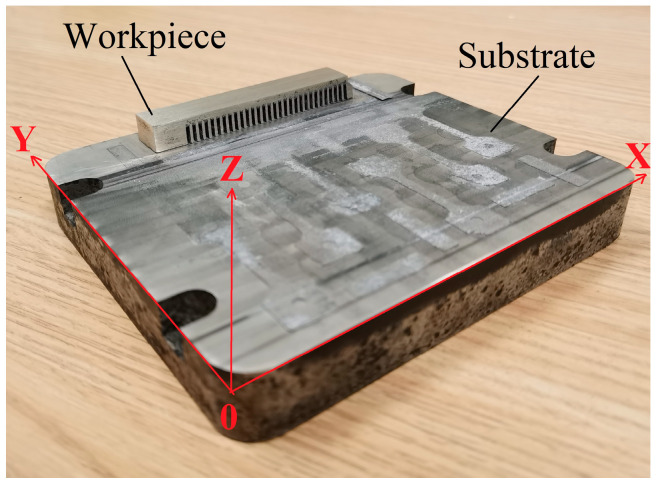
Substrate and scanning direction co-ordinate system of LPBF.

After putting the aforementioned WE43 magnesium alloy powder into the BLT S210 LPBF equipment, argon gas (Ar, with a purity of 99.99%) was circulated. Firstly, a 3D model of the workpiece was designed by utilizing the 3D drawing software (msgics 24) on a computer. With layering software, the model was discretized into layers of data, generating a 2D laser processing trajectory. After inputting the LPBF equipment, the substrate and scanning direction co-ordinate system were printed, as shown in Figure 1. The X–Y surface scanning strategy was as follows: S-shape scanning within one layer and 67° rotation between layers. WE43 powder was laid in advance. The main parameters of scanning included laser power of 80 W, a laser scanning rate of 800 mm/s, and scanning spacing of 70 μm. After scanning each layer, rubber scraper was used to lay another layer of WE43 powder on the finished layer, and the high-energy beam laser was adopted to scan again in accordance with the already set processing trajectory, forming the layer with a thickness of 20 μm. Through back and forth cycling in this way, the layers were stacked (Z direction) until the entire workpiece was completely manufactured.

**Figure 2:**
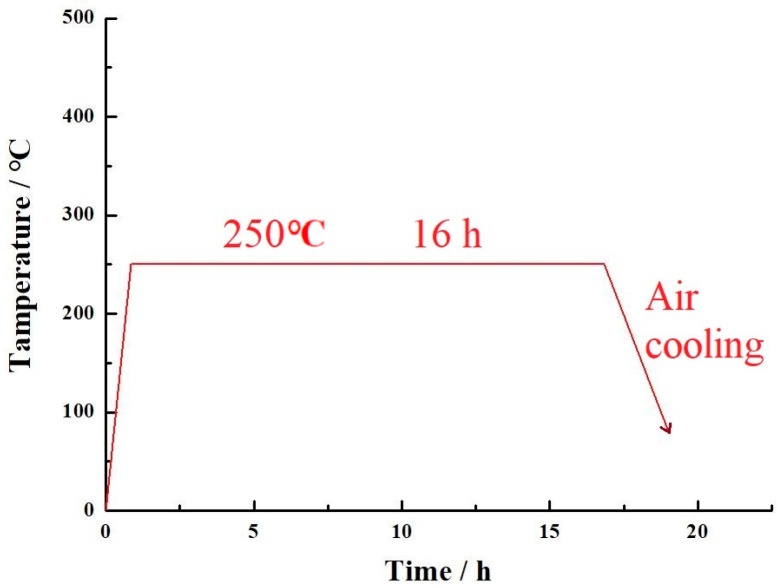
Schematic diagram of T5 aging heat treatment process.

Afterwards, wire-electrode cutting was employed to cut apart the workpiece and substrate. The above workpiece was put into the YTGKD205-11 tubular vacuum heat treatment furnace (vacuum degree of 50 Pa) for T5 aging heat treatment, with a heating rate of 5 °C/min, an aging temperature of 250 °C, and a time of 16 h. After cooling to room temperature, the workpiece was taken out. The aging heat treatment process is shown in Figure 2.

### 2.3. Sample Testing

The test surfaces of the samples were selected to be the surfaces located in the volume of the workpiece and parallel to the cutting direction. With a HV-1000A micro-hardness tester, the hardness of WE43 was tested, revealing the load of 0.2 Kgf and the load holding time of 10 s. For each surface, 5 points on a straight line were selected for testing, taking the average value. Three-dimensional X-ray micro-CT equipment, nano voxel 2000, was used in CT detection and to test of the scale part of sample; the test conditions included voltage 80 kV, current 100 μA, resolution 11.50 μm, and time 1.5 h. By employing the portable μ-X360 full-automatic residual stress detection system manufactured by Pulstec, Japan, the residual stress was tested, with Cr target, collimator diameter of 1 mm, power of 30 KV·1 mA, and zero stress iron powder calibration (calibration error less than 10 Mpa). The microstructure was observed through a field emission scanning electron microscope (FE-SEM, FEI Nova 400 FEG-SEM) and an optical microscope (OM) equipped with electron backscatter diffraction (EBSD) detector. The preparation of samples for EBSD observation involved mechanical polishing and electrochemical polishing using electrolyte AC2. EBSD data were analyzed with commercial software AZtec Crystal 2.1.

## 3. Results and Discussion

### 3.1. Microhardness

**Figure 3:**
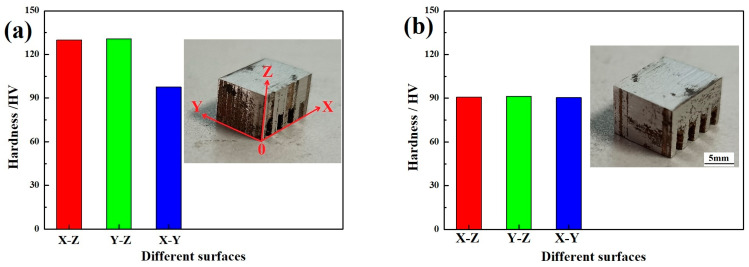
Microhardness HV distribution of WE43 formed by LPBF: (**a**) before aging treatment; (**b**) after T5 aging treatment.

For each of the three respective surfaces of WE43 formed by LPBF, the hardness was tested, with test results as indicated in Figure 3a, before aging treatment; (b) after T5 aging treatment. Before aging treatment, the hardness of the X–Z surface (about 129.9 HV on average) and the Y–Z surface (about 130.7 HV on average) was almost the same, which was approximately 33.5% higher than that of the X–Y surface (about 97.6 HV on average). WE43 formed by LPBF exhibited obvious anisotropy. When testing the hardness, the direction of load application was perpendicular to the tested surface, indicating that the Z direction was the direction with the weaker load-bearing capacity of WE43 formed by LPBF. After T5 aging treatment, the hardness of the X–Y surface (about 90.9 HV on average), the X–Z surface (about 91.4 HV on average), and the Y–Z surface (about 90.4 HV on average) was almost the same, the anisotropy disappeared, and the overall average hardness decreased by 6.8% compared with the X–Y surface before aging treatment. (Due to the scanning strategy of 67° rotation between layers adopted in the experiment, the hardness of the X–Z surface and the Y–Z surface was roughly the same, so the Y–Z surface and the X–Y surface have been selected for comparative study below).

### 3.2. Defects

**Figure 4:**
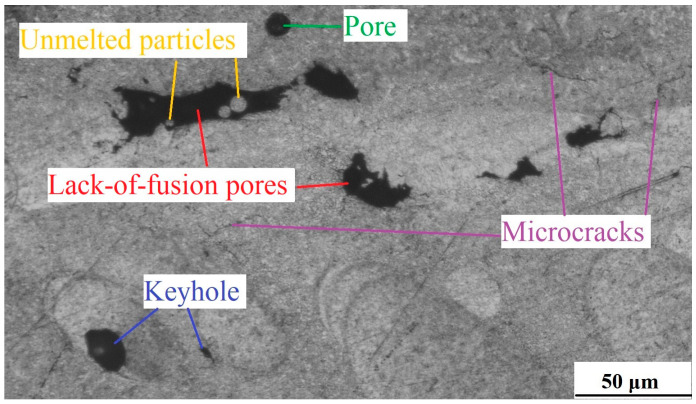
Defects of WE43 formed by LPBF.

During LPBF, some defects will inevitably occur, directly affecting the density and mechanical properties of parts. Figure 4 exhibits the defects of WE43 formed by LPBF, mainly including unmelted particles, pores, unfused holes, keyholes, and cracks.

The pores generally occur under the input of high energy density. At this time, the molten pool has a high temperature, and the low boiling point and high saturated vapor pressure of Mg aggravate the evaporation, producing more Mg vapor, which generates recoil pressure on the molten pool. Due to the huge temperature gradient in the molten pool, a strong Marangoni convection effect forms inside the molten pool. As the depth of the molten pool is much greater than its width, keyhole melting mode occurs. Moreover, the solidification rate is very fast during LPBF, so more Mg vapor cannot escape from the molten pool in a timely manner, thus it exists inside the molten pool in the form of pores [24]. Unfused defects are usually generated under the input of lower energy density. Such defects as irregularly shaped voids between scanning passes or layers can be completely avoided by increasing the laser energy density. But, in fact, even if nominally sufficient laser energy is utilized, the evaporation products may lead to attenuation of the energy transferred by the laser to the powder bed. Moreover, the powder denudation and spatter redeposition around the scanning passes can cause an uneven thickness of the powder layer. In addition, fluctuations in laser energy input and layer thickness will still result in the formation of unfused defects [25]. Cracks may occur in both phases of liquefaction and solidification. Liquation cracks appear in the partial melting zone, where the heat effect exceeds the eutectic temperature. Due to the liquefaction of the grain boundary, the strength of the partial melting zone is weakened. When the solidification shrinkage and thermal shrinkage inside the molten pool exceed the strength of the partial melting zone, cracks may appear. Occurring in the heat-affected zone outside the molten pool, liquation cracks belong to grain boundary cracking, with the fracture surface not showing dendrite morphology. Solidification cracks are generated in the last stage of solidification. At this time, the grains are fully grown, and there are liquid films on the grain boundaries, roughly appearing in the middle of the molten pool. Along with the cracking of grain boundaries, the fracture surface presents dendrite morphology [26].

**Figure 5:**
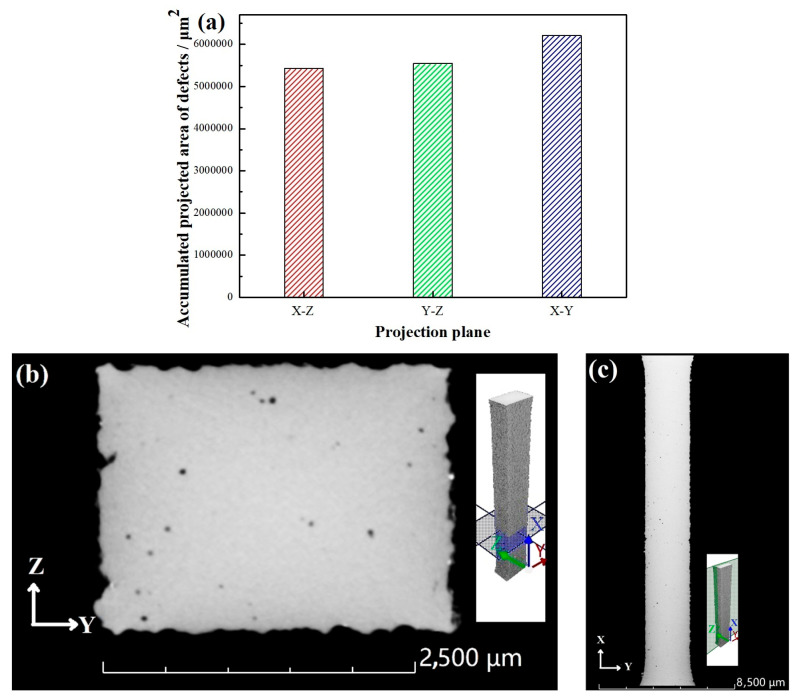
Testing results of void defects: (**a**) cumulative projected area of defects on each surface; (**b**) distribution of defects on Y–Z surface 5.68 mm away from axis 0 in the X direction; (**c**) distribution of defects on X–Y surface 0.88 mm away from axis 0 in the Z direction.

For the uniform distribution of defects on the three surfaces, or otherwise, Figure 5 illustrates the 3D microscopic CT testing results of WE43 formed by LPBF. A total of 2868 void defects were scanned. The cumulative projected area of defects on the X–Z surface (5.43 × 10^6^ μm^2^) was similar to that on the Y–Z surface (5.54 × 10^6^ μm^2^), and the projected area of defects on the X–Y surface (6.21 × 10^6^ μm^2^) was about 13.2% higher than the average of the first two. Generally, the strength and porosity of materials can be expressed as [27]:

(1) $$\sigma_{b}=\sigma_{0}exp\;(-np)$$

where, $\sigma_{0}$ denotes the strength when the porosity is 0, p refers to the porosity, and n is a constant. As observed, there is an exponential function relationship between material strength and porosity, and the material strength will be greatly reduced with an increase in its porosity. When the material is subjected to load, on the one hand, stress concentration is easy to occur near the defect, thus forming the crack source. On the other hand, the cross section area of load can be reduced due to the existence of void defects, thereby reducing material strength. Therefore, the Z direction is the direction with the weaker load-bearing capacity of WE43 formed by LPBF. The distribution of defects is vital to the anisotropy of materials.

### 3.3. Residual Internal Stress

The residual stress test results of WE43 are exhibited in Table 2, where “+” indicates tensile stress and “−” denotes compressive stress. The sample formed by LPBF presents greatly differing participating stresses in various directions, indicating obvious anisotropy. The largest absolute value of residual stress shows in the Z direction, manifested as compressive stress. After T5 aging treatment, the residual stress decreases, and the stresses in various directions are the same and are exhibited as compressive stresses.

**Figure 6:**
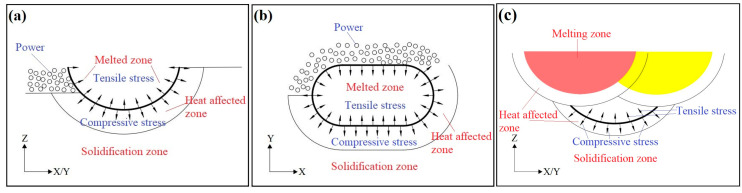
Schematic diagram of residual internal stress analysis of solidification molten pool: (**a**) X/Y–Z surface; (**b**) X–Y surface; (**c**) X/Y–Z surface at re-heat input.

Using 2D graphs, the process of internal stress generation and the accumulation of LPBF are analyzed and illustrated in Figure 6. When the magnesium alloy powder is irradiated by the laser, three zones are formed, namely the melting zone, the heat-affected zone, and the solidified zone. When the laser leaves the melting zone for cooling, the solidification starts from the supercooling zones, such as molten pool boundaries. Hence, cooling shrinkage occurs in the volume, and tensile stress is generated in the melting zone due to the constraint of the heat-affected zone, with the direction consistent with the solidification direction. Considering the high coefficient of thermal expansion of WE43 (about 25~26 μm/°C), there is larger tensile stress in this part. Although the heat-affected zone is in a solid state, it may also be affected by thermal expansion and contraction. As the temperature change in this zone is smaller than that in the melting zone, the compressive stress generated is also less. The contraction direction is from the high temperature zone to the low temperature zone, which is consistent with the stress direction [28,29]. The simplified diagram of the distribution of residual internal stress in solidification is as shown in Figure 6a,b. It is assumed that the distribution of residual stress is uniform in the melting zone and the heat affected zone, shown by arrows. According to stress analysis, the residual stress in the X and Y directions is greater than that in the Z direction.

When the laser passes through the next time, the last melting zone will melt again to reduce the unfused voids. Due to the remelting, the stress on the original X–Y surface will be weakened substantially, and the stress on the X/Y–Z surface will also be redistributed due to the heat input. As exhibited in Figure 6c, the yellow and red zones refer to the new molten pools generated in this laser scanning, with stress distribution the same as that in Figure 6a,b. However, there is still the residual stress in the solidified zone formed the last time, as indicated by the black part in Figure 6c. The tensile stress is sharply reduced due to the overlap of the heat-affected zone, while the compressive stress shows little decrease. According to the stress analysis, the residual stress in the Z direction is greater than that in the other directions, mainly expressed as compressive stress. Similarly, the residual stress accumulates many times through layer-by-layer deposition until the workpiece is complete. The residual stress in the Z direction is far greater than that in the X and Y directions, and the part in the Z direction is the weaker part that can only withstand small loads. Hence, the accumulation of residual stress in the Z direction is one of the main reasons for the anisotropy of LPBF of WE43, resulting in the decline of properties in the Z direction.

During T5 aging heat treatment, the kinetic energy of atoms is increased by temperature, and short-range migration begins. Hence, with the relaxation of the lattice, the elastic strain in the lattice is released, and the secondary phase is gradually precipitated, resulting in the great reduction of residual stress. At the temperature of 250 °C, the energy provided is limited. The high lattice distortion energy shows a quick release rate, while the low lattice distortion energy exhibits a slow release rate. After 16 h, the residual stress tends to be the same in various directions, but cannot completely disappear.

### 3.4. Microstructure

**Figure 7:**
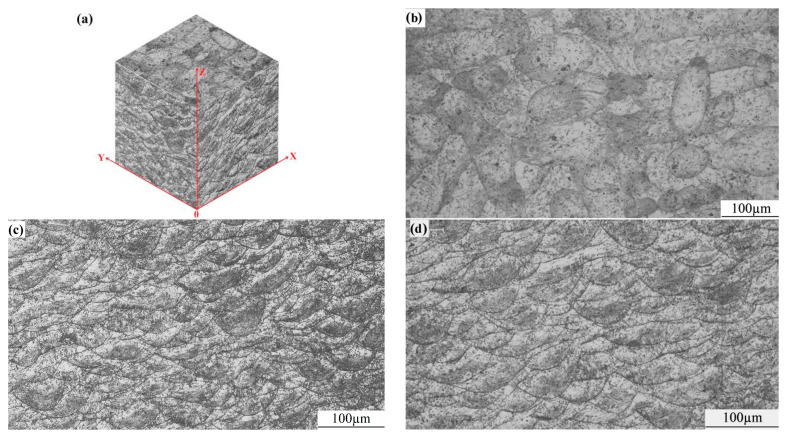
Microstructure of WE43 formed by LPBF: (**a**) 3D morphology; (**b**) X–Y surface; (**c**) X–Z surface; (**d**) Y–Z surface.

Figure 7 presents the microstructure of WE43 formed by LPBF, as shown in (a) 3D morphology, (b) X–Y surface, (c) X–Z surface, and (d) Y–Z surface. The scanning strategy of 67° rotation between layers was adopted in the experiment, which could reduce the residual stress and plastic deformation by decreasing horizontal and vertical cyclic heat input, effectively restraining the generation and propagation of cracks [30]. With this scanning strategy, the scanning paths may not completely coincide in different layers. The X–Z surface and the Y–Z surface exhibit almost the same microstructure, like fish scales. For the X–Y surface parallel to the scanning direction, the microstructure is elliptical.

**Figure 8:**
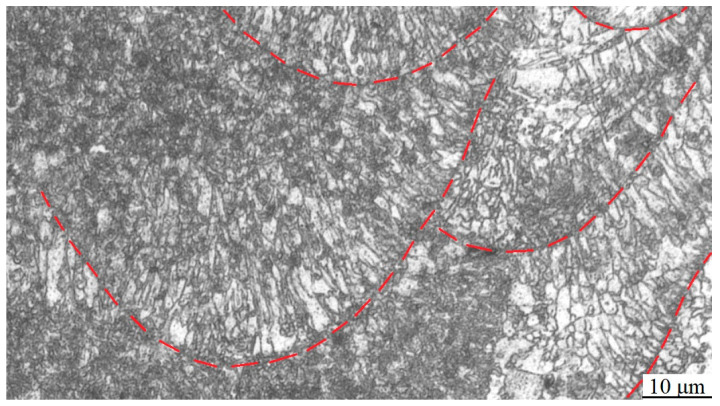
Microstructure of molten pool.

The formation of fish-scale and elliptical structures is essentially related to the growth morphology and distribution of grains in the molten pool. The laser heat source exhibits Gaussian distribution [31]. During static irradiation, the distribution is circular on the irradiation surface and semi-elliptical in the thickness direction of the workpiece. When the laser moves forward, the elliptical molten pool appears on the irradiation surface. The bottom of the molten pool is semi-circular in the cross-section of the moving direction, and a wavy line appears in the moving direction. After the laser heat source leaves, the molten pool shows rapid cooling, solidification, and crystallization. In the center of the molten pool, the grain growth rate is relatively slow, the center temperature is extremely high, with a positive temperature gradient due to the Gaussian distribution of laser energy, the ratio of G/R (temperature gradient/growth rate) is great, and the grains are equiaxed grains. At the boundaries of the molten pool, the melt nucleates with the solid phase at the bottom of the molten pool, growing rapidly and releasing a large amount of latent heat from crystallization, resulting in a negative temperature gradient, and the G/R ratio is small. The grains are dendrites and exhibit reverse selective growth along the heat dissipation direction (perpendicular to the bottom boundary of the molten pool, α = 90°), with a length much greater than the diameter. Thus, along the molten pool boundary (fish-scale and elliptical), fine and long grains grow (Figure 8, the red lines are the boundary of the molten pools.), which are perpendicular to the tangent boundary line [32].

The solid–liquid interface at the bottom of the molten pool is an isothermal line, and theoretically the supercooling degree ∆T is the greatest here. According to the classical solidification principle, the relationship between the critical nucleus radius $r_{k}$ and the supercooling degree ∆T is as follows [33]:

(2) $$r_{k}=\frac{2\sigma }{∆G_{r}}=\frac{2\sigma T_{m}}{L_{m}\cdot ∆T}$$

where, σ denotes the interface energy between the crystal nucleus and the liquid phase, $∆G_{r}$ represents the change in volume of free energy, $L_{m}$ indicates the latent heat of crystallization, $T_{m}$ refers to the melting point, and ∆T means the supercooling degree. As known from Formula (2), the greater supercooling degree corresponds to the smaller critical radius of crystal nucleus, so the smallest grains exist in the bottom boundary of the molten pool.

According to the Hall–Petch formula, the material strength and the grain size and content conform to the following relationship [34]:

(3) $$\sigma_{s}=\sigma_{0}+kd^{-1/2}$$

where, $\sigma_{s}$ refers to the yield strength, $\sigma_{0}$ and k are constants, and d denotes the grain size. The smaller the grain diameter d, the higher the yield strength. Hence, the strength is highest at the bottom boundary of the molten pool.

As known from Figure 7, there are far more molten pool boundaries on the X–Z and Y–Z surfaces compared to those on the X–Y surface. Thus, the X–Z and Y–Z surfaces are distributed with more fine grains, resulting in higher strength by contrast to the X–Y surface. The fine grain-strengthening mechanism also contributes to the anisotropy of WE43 formed by LPBF.

**Figure 9:**
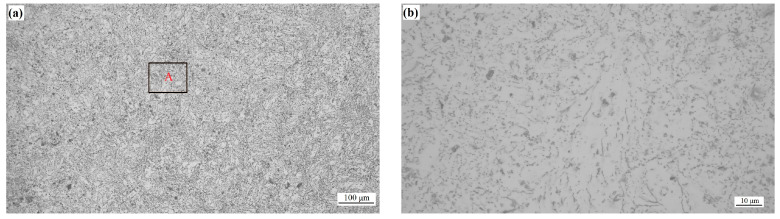
Microstructure after T5 aging treatment; (**b**) representing the enlarged view of zone A in (**a**).

Figure 9 presents the microstructure of WE43 formed by LPBF after T5 aging treatment. The elliptical and fish-scale structures disappear, and the matrix phase α-Mg grows to a certain extent. The amount of the precipitated secondary phase increases slightly, while there is still a great deal of supersaturated solid solution. The structure is relatively uniform, belonging to normal growth.

Due to the low aging temperature, α-Mg involves no phase transition or re-nucleation, and Mg atoms diffuse through the process driven by a decrease in free energy. Firstly, the internal defects of grains exhibit the smallest energy, and Mg atoms rapidly diffuse along the defects, resulting in an ordered atomic arrangement and solute atoms moving to the grain boundaries. Afterwards, Mg atoms diffuse at grain boundaries, and the finer grain size corresponds to the stronger atomic diffusion ability. As the interface moves, the surrounding grains are swallowed, causing the α-Mg phase to grow and more solute atoms to gather at the grain boundaries. At the boundary between elliptical and fish-scale patterns, the diffusion rate is the fastest due to the smallest grain size and the most crystal defects. In the center of the molten pool, the diffusion rate is slower for the larger grains, and there are fewer defects. After 16 h, the microstructure is relatively uniform, and the elliptical and fish-scale patterns disappear.

The nucleation of the secondary phase often occurs at crystal defects, where the high energy provides necessary conditions for nucleation. Under the driving force of phase transition $∆G_{v}$, solute atoms undergo remote diffusion. Due to the lower aging temperature (250 °C), the minor driving force $∆G_{v}$, the lesser diffusion coefficient $D_{\alpha }$, and the slower growth rate, the newly precipitated secondary phase exhibits a smaller volume [35].

### 3.5. Grain Orientation

**Figure 10:**
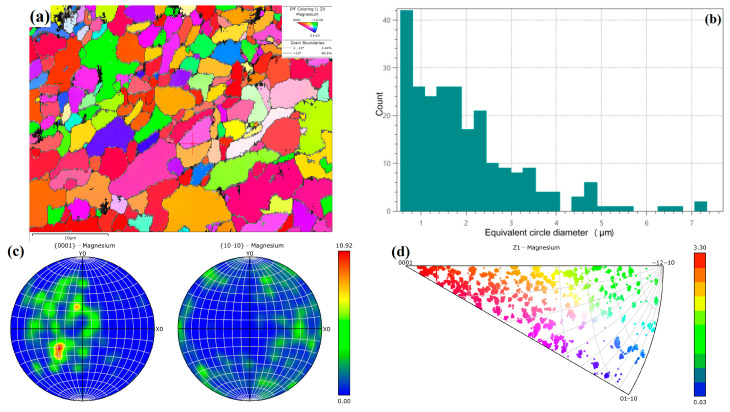
EBSD testing results of WE43 formed by LPBF: (**a**) grain morphology; (**b**) grain size statistics; (**c**) pole figure; (**d**) inverse pole figure.

Figure 10 exhibits the EBSD testing results of grain morphology of WE43 formed by LPBF. During the formation by LPBF, due to the small spot diameter and molten pool volume, the cooling rate is very quick during solidification, and the supercooling degree ΔT is large. In this extremely fast non-equilibrium solidification process, the grain size is very small, and numerous nanoscale grains are generated, with an average size of 2.0 μm (Figure 10b). The grain growth direction is related to the heat dissipation direction, and the vertical processing method of LPBF makes most grains grow in the <0001> direction. However, due to the too-fast solidification rate and the obstruction of the secondary phase, there is not enough time for the newly formed nucleus to grow, resulting in a low concentration of growth direction (Figure 10c,d). Simultaneously, the scanning strategy of 67° rotation between layers was adopted in the experiment, thus reducing the horizontal and vertical cyclic heat input. Hence, the grain growth direction exhibits a certain dispersion, even deviating from the <0001> direction somewhat, resulting in a weak texture.

**Figure 11:**
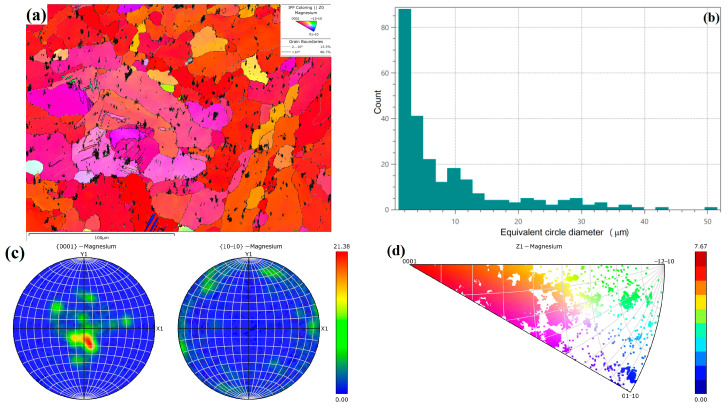
EBSD testing results of the WE43 sample formed by LPBF after T5 aging treatment: (**a**) grain morphology; (**b**) grain size statistics; (**c**) pole figure; (**d**) inverse pole figure.

Figure 11 presents the EBSD testing results of the WE43 sample formed by LPBF after T5 aging heat treatment. During T5 aging treatment, the atomic kinetic energy increases at 250 °C. With outward migration of grain boundary, other grains are absorbed, resulting in obvious growth of the grain size, with an average size of 8.5 μm. The grain orientation has obvious directionality. The strength index of texture orientation increases from 10.92 in the printed state to 21.38, roughly along the <0001> direction. The grain boundaries with the small angle of 2~10° increase from 3.44% to 13.3%, and those with a large angle exceeding 10° decrease from 96.6% to 86.7%. This is inconsistent with the expected results. It is mostly believed that the grain orientation can be dispersed into multiple directions due to the heat treatment, showing no obvious texture.

**Figure 12:**
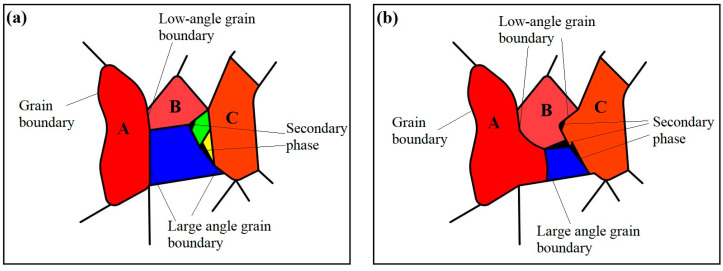
Schematic diagram of grain growth change: (**a**) Initial grain of LPBF WE43; (**b**) Growing grains after T5 aging treatment.

Figure 12 illustrates the process of grain growth change in this experiment. Unlike the growth mode after recrystallization, there is no re-nucleation process, and the growth is directly driven by the reduction of interface energy. Due to the rapid non-equilibrium solidification in the early stage, the grain size is very small. Considering the heat dissipation mode from top to bottom, most of the grains face the <0001> direction. Hence, the embryo takes shape. As shown in Figure 12a, the orientations of the three embryos A, B, and C are close to each other, blue, green, and yellow represent grains with significant differences in orientation, and black is the second phase. The growth of embryo is realized by the migration of large-angle grain boundaries. The curved grain boundary has a tendency to move toward the center of its curvature for straightness. With the gradual movement of grain boundary, the other surrounding grains are absorbed. As known from the grain growth theory, when the grain orientations on both sides of the grain boundary are similar, the migration rate of the grain boundary is small. Therefore, the larger the difference in grain orientation, the faster the migration rate of grain boundary. When the grain boundary migrates to the grain with a similar orientation, the migration rate will be very low, or the migration may even be stagnant. After a period of time, the volume of the grains with the same orientation become larger and larger. Simultaneously, as magnesium alloy has an hcp crystal structure with low symmetry, its atomic diffusion exhibits strong anisotropy at low temperatures [36]. Since the (0001) crystal surface is a densely packed surface of atoms, there is large activation energy for diffusion of solute atoms along this surface. The diffusion coefficient along the (0001) crystal surface is smaller than that along the [0001] crystal direction, so it is easy to form the grains along the <0001> direction. However, the anisotropy of diffusion gradually decreases with the increase in temperature.

During grain growth, a small amount of secondary phase can be precipitated from supersaturated α-Mg due to the release of lattice distortion energy, which hinders the further growth of the grain. The grain growth rate has a direct relation with the temperature, and the average migration rate $\bar{m}$ is directly proportional to $e^{{-s}_{m}/RT}$ ($Q_{m}$ indicates the activation energy for grain boundary migration or for atomic diffusion through grain boundary). As the T5 aging treatment temperature (250 °C) is not high enough to provide higher energy, the grain growth rate and size are limited, as presented in Figure 12b.

According to Schmid’s law [37], when the shear stress $\tau_{s}$ in the slip direction of the slip surface reaches the critical value, the crystal begins to yield, and $\tau_{s}$ and $\sigma_{s}$ satisfy the following relationship:

(4) $$\tau_{s}=\sigma_{s}\cos\lambda \cos\varphi$$

where, $\sigma_{s}$=σ, φ refers to the angle between the normal of the slip surface and the force axis, λ represents the angle between the slip direction and the force axis, andcos⁡λcos⁡φ indicates the orientation factor. As indicated, when λ or φ is 90°, $\sigma_{s}$ is infinite, regardless of the value of $\tau_{s}$, indicating that the material involves no slip deformation under external force.

The atomic arrangement of single crystal magnesium is in an hcp structure. When deformed at room temperature, there is only one slip surface (0001) with a slip direction of <1120> [38]. When the load direction is in the Z direction, the angle λ between slip direction and force axis is 90°, and slip cannot occur. When the load direction is X or Y, the minimum angle λ between slip direction and force axis is 0°. Once the yield strength $\sigma_{s}$ of magnesium is reached, the slip begins. This indicates that the mechanical properties of single-crystal Mg have anisotropy, and the highest strength exists in the <0001> direction.

LPBF can realize solid solution of the multiple rare earth elements and modified elements into the α-Mg matrix with an extremely fast cooling rate, generating a structure with ultra-high solid solubility. Due to the regularity of intervals in crystals, Mg interstitial solid solutions are in octahedra and tetrahedra, both of which are composed of atoms on the slip surface (0001). Both octahedra and tetrahedra have high stability, which makes slipping impossible. By replacing solute atoms in a solid solution, the surrounding lattice distortion can be aroused, further hindering the slipping [39]. Therefore, the structure with ultra-high solid solubility in LPBF WE43 shows no obvious anisotropy.

After T5 aging treatment, the precipitated phase is gradually separated out from the ultra-high solid solubility structure in LPBF WE43. With the increase in α-Mg, the anisotropy grows, namely the strength increases in the <0001> direction. Meanwhile, T5 aging treatment results in a consistent grain orientation, forming a <0001>-oriented texture. The combination of the above two factors greatly strengthens the <0001> direction. Due to its parallel to the load direction Z, the <0001> direction can withstand the maximum load.

In this experiment, most of the grains of WE43 formed by LPBF are in the <0001> direction, but the grain orientation exhibits low concentration. After T5 aging treatment, the grain orientation tends to be the same, forming an obvious texture, accompanied by the disappearance of anisotropy. Studies have shown [40,41,42] that other materials formed by LPBF exhibit obviously weakened texture after heat treatment at a higher temperature, but there is still anisotropy. Therefore, it is speculated that the following reason contributes to the disappearance of anisotropy in this experiment. T5 aging heat treatment cannot effectively reduce the number of defects [43], and there is still a weakening effect on the mechanical properties in the Z direction. However, the texture in the <0001> direction has an enhanced effect on the mechanical properties in the Z direction. As the two effects cancel each other out, the anisotropy is weakened.

## 4. Conclusions

(1) The mechanical properties of WE43 magnesium alloy formed by LPBF exhibit obvious anisotropy. The hardness HV of the X–Z surface (129.9 HV on average) and the Y–Z surface (130.7 HV on average) is about 33.5% higher than that of the X–Y surface (97.6 HV on average), and the endurable load is smaller in the stacking direction compared to in the X and Y directions. After T5 (250 °C/16 h) aging heat treatment, the anisotropy disappears.

(2) The factors contributing more to the anisotropy of mechanical properties of WE43 magnesium alloy formed by LPBF are listed in sequence. Firstly, the defect area of the X–Y projection surface is about 13.2% higher than that of the other two surfaces. Considering the exponential relationship between the material strength and the number of defects, the mechanical properties of this surface are greatly reduced. Secondly, for laser scanning in each layer/time, the residual stress accumulation in the Z direction is higher than that in the X and Y directions, which may directly reduce the mechanical properties of the material. Finally, the smallest grains exist at the solid–liquid interface at the bottom of the molten pool, more fine grains are distributed in the X–Z and Y–Z surfaces when compared to those in the X–Y surface, and this fine grain-strengthening mechanism also contributes to the anisotropy.

(3) After T5 aging heat treatment (250 °C/16 h), a stronger crystallographic texture is formed in the <0001> direction, with the orientation density index increasing from 10.92 to 21.38, and the anisotropy disappearing. This is mainly caused by the fact that the enhancement effect of the texture in the <0001> direction on the mechanical properties in the Z direction cancels out the weakening effect of more defects in the X–Y surface in the Z direction.

## Figures and Tables

**Table 1:** Chemical composition of WE43 magnesium alloy (wt.%).

Element	Zn	Zr	Gd	Nd	Y	Mg
Content	0.21	0.4	1.23	2.46	3.77	Bal.

**Table 2:** Residual stress test results of WE43.

Sample	Test Surface	Residual Stress (MPa)	Residual Stress (MPa)
LPBF WE43	X–Y	+147 (X direction)	−332 (Y direction)
	Y–Z	−165 (Y direction)	−493 (Z direction)
LPBF WE43 T5	X–Y	−150 (X direction)	−146 (Y direction)
	Y–Z	−139 (Y direction)	−145 (Z direction)

## Data Availability

The datasets used and/or analyzed during the current study are available from the corresponding authors on reasonable request.
